# Development of a dietary management indicator system for older adults with disabilities: a Delphi study

**DOI:** 10.3389/fpubh.2026.1796150

**Published:** 2026-03-23

**Authors:** Meiyao Li, Aijuan Zhao, Yang Lin, Jing Shi, Wenwen Chen, Ping Zhang

**Affiliations:** 1 Department of Clinical Medical, Jiamusi University, Jiamusi, Heilongjiang, China; 2 Department of Retirement Work, The First Hospital Affiliated of Jiamusi University, Jiamusi, Heilongjiang, China

**Keywords:** Delphi method, dietary management, disabled older adults, nutritional needs, semi-structured interviews

## Abstract

**Objectives:**

This study aims to develop a national consensus on the needs and dietary management of disabled older adults and guide their nutritional care.

**Methods:**

A systematic review of the literature in the Pubmed was conducted to identify evidence regarding the nutritional management of disabled older adults people. Subsequently, an expert panel was formed to understand their perspectives on the nutritional management of disabled older adults people through semi-structured interviews. Finally, through a modified Delphi method, a multidisciplinary expert panel was invited to refine and validate the nutritional management indicators for disabled older adults people.

**Results:**

The average score of three expert coordination coefficients were 0.95 (judgmental basis), 0.86 (familiarity), 0.90 (authority) respectively. The average score of Kendall’s W was 0.890. A comprehensive list for the nutritional management of disabled older adults people was established, which covers 35 primary indicators, 16 sary indicators, and 33 tertiary indicators.

**Conclusion:**

The index system of nutritional needs and dietary management for disabled older adult individuals, as constructed in this study, provides a theoretically grounded framework that requires further empirical validation. It provides a reference for the nutritional management of disabled older adults in the future.

## 1 Introduction

“Disability” and “Incapacity” are distinct yet interrelated constructs defined by WHO, where disability emphasizes social participation barriers from long-term impairments while incapacity focuses on functional loss affecting self-care. This study targets older adults with functional limitations including illness-, injury-, or congenitally-induced disabilities, whose population is projected to grow from 52.71 million in 2020 to 95.37 million by 2050 in China (1). These individuals face compounded nutritional risks, including protein-energy malnutrition and dysphagia-related complications, which significantly impact quality of life (2, 3).

In China, the living conditions for older adult individuals with disabilities vary significantly depending on geographic location, socioeconomic status, and availability of support services. Traditionally, many older adult individuals with disabilities in China live with their families, often in multi-generational households. This family-centred approach is consistent with Confucian values that emphasise filial piety and family responsibilities (1). In urban areas, there has been a gradual shift toward institutional care due to increased mobility and changing family structures. However, institutional care facilities, including nursing homes and assisted living centers, are still relatively underdeveloped compared to Western standards (4). In rural areas, the availability and quality of institutional care are even more limited, leading many older adult individuals with disabilities to rely on informal family support (5) Compared with the West, dietary management for Chinese older adults with disabilities is more oriented toward home-based dietary management. Often the carer decides on the three meals a day for the cared-for person. Therefore, there is a need to provide programmes for the dietary management of older adults people with disabilities in China.

Agarwal et al. (6) have found that diet may be an effective strategy for preventing dysfunction in older adults. Therefore there is a need to manage the diet of older adults with disabilities. Dietary management refers to the scientific and rational arrangement and management of diets. Reasonable dietary management not only meets the nutritional needs of the organism, maintains health and prevents disease, but also provides the right amount of energy and nutrients to promote health and development. Much literature has focused on dietary management for older people, including the prevention of dementia (7), diabetes (8) and frailty (9) in older adults people. Beyond disease-specific outcomes, nutritional status is closely linked to overall quality of life in older adults with disabilities. Malnutrition has been shown to accelerate functional decline, exacerbate frailty, and increase the risk of depression, thereby diminishing both physical and mental wellbeing (10–12). In fact, the US Centers for Disease Control and Prevention (CDC) has begun to focus on the nutritional management of adults with physical disabilities (13). A study by Chinese scholar Kang Lin also pointed out that appropriate nutritional support and team interventions targeting multiple risk factors can effectively improve the prognosis of disabled older adults (14). However, there are limitations in the nutritional management of older adult individuals with disabilities. Evidence-based guidelines may not be able to address some of the controversial issues that experts face in their daily work. Expert consensus and Delphi studies may be useful tools in addressing this issue (15).

This study was aim to reach a consensus on the principles of constructing dietary management for the disabled older adults and highlight areas for improving nutritional care in managing disabled older adults in China. The objectives of the study were as follows: (1) to reach an expert consensus on the principles of dietary management for the disabled older adults in China; and (2) to gather information on the areas of highest uncertainty related to the nutritional management of the clinically disabled older adults.

## 2 Materials and methods

### 2.1 Design

The Delphi method is widely recognized as a powerful means for respondents to reach a consensus and generate ideas on many health-related issues. Therefore, considering the low available evidence, knowledge, or application, this study used a Delphi method to explore expert opinion on dietary guidelines for older adults with disabilities. Delphi is a feedback-based anonymous questioning technique. After obtaining the opinion of the experts on the question to be asked, it is statistically analyzed and then anonymously fed back to the experts until a consensus opinion is obtained. Three main aspects are ensured in this study: (1) the experts do not know the identity of the other experts, which helps to ensure that their responses are independent; (2) the experts answered individually to avoid certain individuals dominating the group; (3) a mathematical voting procedure was used to rank the items. This independent consultation ensures that experts do not discuss or exchange opinions.

#### 2.1.1 Step 1: literature review

**Figure 1:**
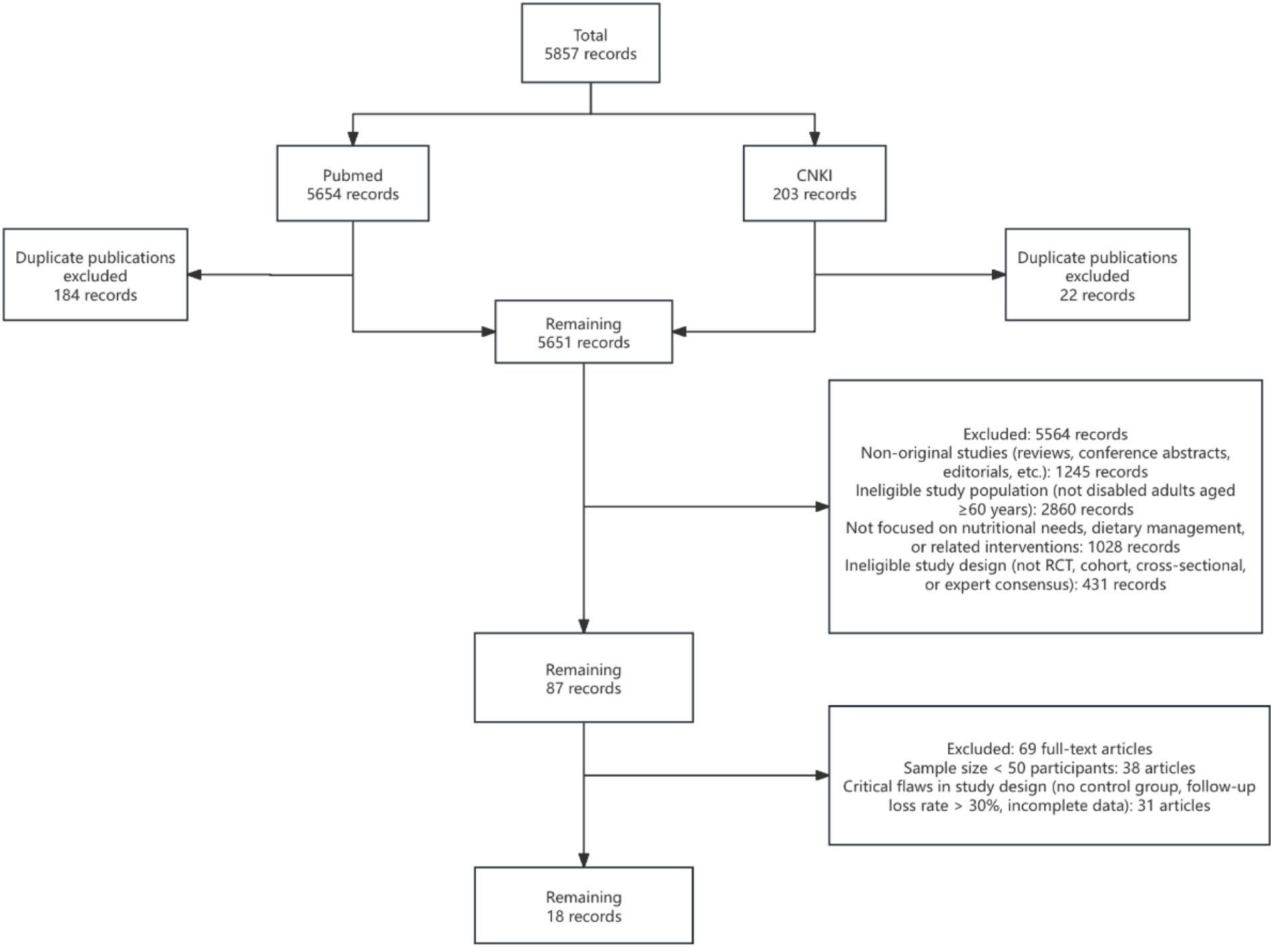
PRISMA flow.

We reviewed the literature in Pubmed and CNKI from 2015 to 2025 based on the keywords (((((((persons with disabilities) AND (older adults)) AND (Nutrition status)) OR (Nutrition Assessment)) OR (Nutrition Surveys)) OR (Nutritional Requirements)) OR (Energy Intake)) AND (Mental health). Inclusion Criteria: (1) The research subjects are disabled or incapacitated older adults people aged 60 and above. (2) The study involves nutritional requirements, dietary management, or related intervention measures. (3) The research types include randomized controlled trials, cohort studies, cross-sectional surveys, or expert consensuses. (4) The literature is published in core journals. Exclusion Criteria: (1) Duplicate publications. (2) Non-original research (such as reviews and comments). (3) Studies with a sample size of <50 cases. (4) Research designs with major flaws (such as no control group or a follow-up loss rate of more than 30%). We followed the Preferred Reporting Items for Systematic Reviews and Meta-Analyses (PRISMA) guidelines to ensure transparency in our literature search and selection process. Study quality was assessed using the Newcastle-Ottawa Scale (NOS) for cohort studies and the Cochrane Risk of Bias tool for randomized controlled trials, ensuring only high-quality studies were included. Two reviewers independently screened titles, abstracts, and full texts using predefined inclusion/exclusion criteria, with disagreements resolved by a third reviewer. Eventually, 18 articles were included (16–34) (Figure 1).

#### 2.1.2 Step 2: qualitative phase—semi-structured interviews

The second phase involved semi-structured interviews with clinical experts in geriatric nursing from the emergency departments of three tertiary hospitals. Guided by the Symptom Management Model (33), these interviews explored the participants’ perspectives on the dietary management indicators for disabled older adult individuals and the application of dietary management principles in clinical practice. The interviews adhered to the Consolidated Criteria for Reporting Qualitative Research (COREQ) guidelines, with a topic guide developed by two trained researchers with expertise in geriatric nursing. Interviews were audio-recorded, transcribed verbatim, and analyzed using reflexive thematic analysis, with codes cross-validated by a third researcher to ensure trustworthiness. Participant characteristics (e.g., years of experience, professional roles) were documented, and quotes were anonymized to protect confidentiality. Key topics included symptom recognition These findings provided the basis for the initial draft of the nursing - sensitive quality indicators specific to disabled older adult individuals, which were subsequently refined through the Delphi consensus process.

#### 2.1.3 Step 3: delphi phase—consensus building and interaction analysis

Based on the results of the literature review and qualitative interviews, the third phase employed a modified Delphi method to transform emerging themes into consensus - based priorities (35). A multidisciplinary panel of 16 experts was purposively selected based on: (1) ≥ 10 years of clinical or academic experience in geriatric nursing, nutrition, or rehabilitation; (2) active involvement in the care of disabled older adults; (3) willingness to participate in two rounds. Experts were recruited from tertiary hospitals (*n =* 12), community health centers (*n =* 2), and academic institutions (*n =* 2) across five provinces in China, ensuring geographic and institutional diversity. All 16 experts completed both rounds, yielding a 100% response rate. In each round, Each questionnaire included explicit definitions for all indicators. Subjective terms such as “adequate,” “appropriate,” and “sufficient” were operationalized based on established dietary guidelines. Specifically: “Adequate vegetables and fruits” was defined as ≥300 g of vegetables and ≥200 g of fruits per day, in accordance with the Dietary Guidelines for Chinese Residents 2022. “Appropriate intake of fats and sugars” was defined as total fat contributing ≤30% of daily energy intake and added sugars ≤10%, following WHO recommendations. “Sufficient water intake” was defined as ≥1.5 L/day for older females and ≥1.7 L/day for older males, adjusted for climate and physical activity levels. “Appropriate staple food intake” was defined as 200–300 g of cooked rice or equivalent per meal, based on Chinese geriatric nutrition guidelines. These definitions were included in the Delphi questionnaires and explained to experts prior to scoring to ensure consistent interpretation.” Consensus thresholds were defined *a priori* based on established Delphi methodology: (1) mean score (M) ≥ 3.50 on a 5-point Likert scale; (2) full-score rate (FR) ≥ 20%; (3) coefficient of variation (CV) < 0.25. Indicators meeting all three criteria were retained; those failing any criterion were revised or removed. In each round, a dedicated feedback section prompted experts to elaborate on scores <4, including suggested revisions or conceptual clarifications. Discrepant opinions (CV > 0.3) were summarized in a comparative matrix and redistributed in the next round with anonymized expert rationales, facilitating informed reassessment (36). Anonymity was maintained throughout the Delphi process to minimize hierarchical bias and ensure equitable participation (37). The Delphi process was terminated after two rounds as all indicators met the predefined thresholds and Kendall’s W coefficients demonstrated significant expert harmonization (*p* < 0.05) across all indicator levels. The reporting of this Delphi study adheres to the Conducting and REporting DElphi Studies (CREDES) checklist, ensuring transparency in expert selection, consensus definition, and feedback processes. This process resulted in a validated set of nursing-sensitive quality indicators for the dietary management of disabled older adults.

### 2.2 Setting and sample

The study employed purposive sampling in two phases. In the semi - structured interview phase, five clinical experts, including two nurses and three doctors, were recruited from the emergency departments and nutrition departments of tertiary hospitals in China. They all have rich experience in the care of disabled older adults people. The inclusion criteria included expertise in the dietary management of disabled older adults people, clinical roles, and familiarity with nursing - sensitive quality indicators. In the Delphi consensus phase, a multidisciplinary panel of 16 experts was purposively selected to ensure diversity (Table 1): five nursing experts (including 2 community nursing specialists), five medical experts (3 geriatricians, 2 intensivists), six nutrition experts (2 clinical dietitians, 2 public health nutritionists), and one rehabilitation therapist. Experts were recruited from tertiary hospitals (12), community health centers (2), and academic institutions (2) across five provinces, balancing urban–rural representation. The members of the Delphi panel came from different healthcare settings, covering academic medical centers and regional hospitals, which ensured that different clinical practices and institutional backgrounds were represented.

**Table 1:** Demographic information of participants.

Variables		Frequency (*N*)	Proportion (%)
Age	<45	6	34.7
	45–55	8	48.6
	>55	2	16.7
Sex	Male	9	56.3
	Female	7	43.7
Education degree	Undergraduate	3	20.8
	Master’s	8	47.2
	Doctorate	5	31.9
Work experience	10–20	10	59.7
	>20	6	40.3
Title	Intermediate	11	66.7
	Advanced	5	33.3
Current address	Heilongjiang	5	30.8
	Shenyang	4	23.9
	Beijing	2	12.5
	Guangzhou	2	13.9
	Xi’an	3	18.9

### 2.3 Statistical analysis

The qualitative data obtained from semi-structured interviews were analyzed using the Reflexive Thematic Analysis (RTA) method (38). This iterative process emphasizes the dynamic interaction among researchers, data, and theoretical frameworks. Following the six - phase approach of RTA, the audio recordings were transcribed verbatim, and systematic analysis was conducted through repeated immersion in the transcripts to generate codes, construct themes, and refine interpretations (39). The codes derived from participants’ descriptions of clinical challenges were mapped to the domains of the Symptom Management Model. Potential themes were rigorously reviewed against the original data and contextualized within the dimensions of dietary management care for disabled older adults people. Ambiguities were resolved through reflexive discussions. The final themes provided the basis for the development of dietary management indicators for disabled older adults people.

The Delphi method was used to develop sensitive indicators for evaluating the dietary management of disabled older adults people. Questionnaires were distributed to experts in the field, and data analysis was performed using EXCEL 2019 and SPSS 26.0 software. The expert questionnaires evaluated each indicator based on importance, rationality, and feasibility using a 5 - point scale. Higher scores indicated greater expert recognition. Three criteria were established for retaining items: mean score (M) ≥ 3.50, full-score ratio (FR) ≥ 20%, and coefficient of variation (CV) < 0.25 for all indicators. The Delphi process was terminated after two rounds as all indicators met the above criteria, and Kendall’s W coefficients for expert harmonization were statistically significant (*p* < 0.05) in both rounds, indicating stable consensus. Meeting all three criteria indicated a strong consensus and recognition among experts for that item, confirming its significance within the system.

## 3 Results

Through a systematic literature review and thematic analysis of semi-structured interviews, a three-tiered framework for nursing quality indicators for disabled older adults people was established. The literature review identified key areas, including four interrelated dimensions: the nutritional intake dimension, the family care dimension, the medical support dimension, and the psychological and social factors dimension. The interviews also uncovered implementation barriers, including delays in the nutritional management of disabled older adults people by the family support system and inconsistent implementation of medical institution interventions. At the same time, facilitators such as the support of psychological state and social support for nutritional management were also identified. By synthesizing these insights, the preliminary framework was refined into a Delphi consultation scale with 5 first-level items, 16 second-level items, and 33 third-level items, ensuring the consistency between empirical evidence and clinical practice.

### 3.1 Experts’ enthusiasm and authority coefficient

In this study, 16 questionnaires were distributed to experts in relevant fields during both rounds of consultation. All 16 questionnaires were valid, and the validity rate reached 100% in both rounds. The analysis of expert coordination reveals several distinct and statistically significant patterns regarding the harmonization of expert opinions across different hierarchical levels and indicator complexities (Table 2).

1. Hierarchical effect on consensus strength

**Table 2:** Results showing the effects of coordination between experts on opinions and coefficient of harmonization of expert opinions.

Hierarchical level	Indicator Set	Coefficient of experts’ judgmental basis (Ca)	Coefficient of experts’ familiarity (Cs)	Coefficient of experts’ authority (Cr)	Kendall’s W	$\chi^{2}$	*P*
First-Level	5	0.95	0.85	0.90	0.849	13.854	0.030
Second-Level	5	0.98	0.88	0.93	0.916	14.656	<0.001
First-Level	11	0.93	0.83	0.88	0.855	13.680	0.032
Second-Level	11	0.95	0.87	0.91	0.960	15.360	<0.001
First-Level	43	0.91	0.82	0.865	0.843	13.488	0.032
Second-Level	43	0.97	0.89	0.93	0.915	14.640	<0.001

A consistent and primary finding is the stronger consensus observed at the second hierarchical level compared to the first level. For every indicator set (5, 11, and 43), all three coordination coefficients—judgmental basis (Ca), familiarity (Cs), and authority (Cr)—as well as Kendall’s W concordance coefficient, are markedly higher at the second level. For instance, with 43 indicators, Ca increases from 0.91 (First-Level) to 0.97 (Second-Level), and Kendall’s W rises from 0.843 to 0.915. This demonstrates that expert opinion convergence is more robust when evaluating detailed, sub-level criteria than broader, top-level categories.

2 Impact of indicator system complexity

The data delineate a clear influence of indicator quantity on coordination. At the First-Level, increasing the number of indicators from 5 to 43 leads to a measurable decrease in the Ca, Cs, and Cr coefficients (e.g., Ca drops from 0.95 to 0.91), suggesting that greater complexity marginally challenges initial consensus formation on broad criteria. In contrast, at the Second-Level, these coefficients exhibit notable resilience or even improvement; Ca and Cr for 43 indicators (0.97 and 0.93, respectively) remain comparable to or exceed values for simpler sets. This indicates that while complexity may slightly dilute broad-level agreement, it does not undermine—and may even reinforce—the consensus on specific, detailed assessments.

3 Relative performance of coordination dimensions

Among the three coordination dimensions, a stable hierarchy is evident: the coefficient for judgmental basis (Ca) is consistently the highest across all conditions, followed by authority (Cr), with familiarity (Cs) being the lowest. This pattern implies that experts achieve the strongest alignment in their fundamental logical reasoning and methodological approaches, a moderately strong alignment in their perceived authority on the topics, and the relatively weakest, though still substantial, alignment in their self-rated familiarity.

4 Statistical robustness of consensus

The statistical significance of the observed consensus is uniformly high. All Kendall’s W values are substantial (ranging from 0.843 to 0.960), and the associated chi-squared tests are statistically significant (*p* < 0.05 or *p* < 0.001), confirming that the measured agreement among experts is unlikely to be due to chance.

In summary, the coordination of expert opinions is systematically influenced by both the hierarchical depth and the complexity of the indicator framework. Consensus is significantly stronger at more detailed levels of analysis. Furthermore, expert alignment is most pronounced in the domain of judgmental rationale and remains statistically robust even as the number of indicators increases, particularly within detailed hierarchical evaluations. These findings underscore the importance of hierarchical structuring in expert elicitation processes and highlight the differential contributions of judgment basis, authority, and familiarity to overall opinion harmonization.

### 3.2 First round Delphi results

The preliminary evaluation of primary indicators showed a high degree of consensus validity among experts. The appropriateness scores ranged from 4.78 to 4.93 (CV = 0.12–0.24; FR = 86.0–88.6%). The importance scores demonstrated a stronger consensus (M = 4.75–5.00, CV = 0.00–0.35), with FR ranging from 79.3 to 100%. The operational feasibility scores were consistent with the appropriateness metrics (M = 4.74–4.91, CV = 0.18–0.34; FR = 79.0–93.80%). All primary indicators met the inclusion criteria and were retained without modification (Table 3). Therefore, there was no need for a second round of expert consultation on the primary indicators.

**Table 3:** First-round expert consultation results for primary indicators.

Primary indicators	Applicability				Importance				Operational feasibility			
	M	SD	CV	FR (%)	M	SD	CV	FR (%)	M	SD	CV	FR (%)
1. Nutritional Intake	4.78	0.21	0.04	80.0	4.81	0.33	0.07	85.6	4.81	0.24	0.05	85.6
2. Family arrangement	4.89	0.24	0.05	87.3	5.00	0.00	0.00	100.0	4.78	0.30	0.06	80.0
3. Medical Support	4.93	0.24	0.05	94.6	4.78	0.35	0.07	80.0	4.91	0.34	0.07	93.8
4. Mental condition	4.86	0.12	0.02	86.5	4.75	0.21	0.05	79.3	4.74	0.18	0.04	79.0
5. Nutritional status	4.83	0.16	0.16	86.0	4.78	0.03	0.01	80.0	4.88	0.23	0.05	87.0

M, mean; SD, standard deviation; CV, coefficient of variation; FR, full score rate.

For secondary indicators, broad consensus has been reached in terms of appropriateness (M = 4.65–4.95, CV = 0.12–0.48; FR = 85.5–95.6%), importance scores (M = 4.78–4.94, CV = 0.13–0.26; FR = 80.0–94.5%), and operational feasibility (M = 4.73–4.94, CV = 0.03–0.28) (Table 4).

**Table 4:** First-round expert consultation results for secondary indicators.

Secondary indicators	Applicability				Importance				Operational feasibility			
	M	SD	CV	FR (%)	M	SD	CV	FR (%)	M	SD	CV	FR (%)
1.1. Types of food	4.80	0.14	0.03	85.5	4.86	0.13	0.03	86.5	4.83	0.12	0.02	86.1
1.2. Intake amount	4.90	0.03	0.01	93.6	4.88	0.14	0.03	87.0	4.94	0.05	0.01	95.5
1.3. Eating frequency	4.84	0.10	0.02	86.2	4.94	0.05	0.01	88.8	4.73	0.22	0.05	78.9
2.1. Family diet arrangement	4.65	0.18	0.04	86.3	4.88	0.19	0.04	87.0	4.85	0.18	0.04	86.3
2.2. The knowledge and skills of caregivers	4.88	0.05	0.01	87.0	4.94	0.05	0.01	88.7	4.83	0.22	0.05	86.0
2.3. Family support system	4.88	0.14	0.03	87.0	4.93	0.06	0.01	94.2	4.81	0.10	0.02	85.6
3.1. Medical Intervention	4.90	0.05	0.01	93.7	4.93	0.07	0.01	94.2	4.79	0.28	0.06	80.2
3.2. Implementation of Nutritional Support	4.95	0.03	0.01	95.6	4.78	0.13	0.03	80.0	4.86	0.13	0.03	86.4
3.3. The accessibility of medical resources	4.85	0.16	0.03	86.2	4.83	0.16	0.03	86.0	4.80	0.03	0.01	85.5
4.1. Mental condition	4.83	0.15	0.03	86.0	4.87	0.23	0.05	86.7	4.89	0.13	0.03	89.0
4.2. Social Support	4.85	0.12	0.02	86.2	4.83	0.21	0.04	86.0	4.90	0.06	0.01	93.6
5.1. Nutritional status	4.86	0.16	0.03	86.5	4.89	0.18	0.04	89.0	4.92	0.07	0.01	94.1
5.2. Health condition	4.89	0.13	0.03	89.0	4.91	0.09	0.02	94.0	4.90	0.09	0.02	93.6
5.3. Functional status	4.92	0.06	0.01	94.1	4.80	0.20	0.04	85.5	4.85	0.18	0.04	86.2

M, mean; SD, standard deviation; CV, coefficient of variation; FR, full score rate.

Among the tertiary indicators, except for the indicators 2.1.1, 3.2.3, and 5.1.1, they have reached the consensus thresholds in terms of appropriateness (M = 4.75–5.00, CV = 0.00–0.14; FR = 81.10–100.00%) and operational feasibility (M = 4.83–5.00, CV = 0.00 = 0.04; FR = 81.5–100.0%). The importance scores (M = 4.79–5.00, CV = 0.00–0.07) have also reached a consensus (Table 5).

**Table 5:** First-round expert consultation results for tertiary indicators.

Tertiary indicators	Applicability				Importance				Operational feasibility			
	M	SD	CV	FR (%)	M	SD	CV	FR (%)	M	SD	CV	FR (%)
1.1.1 Is the variety of foods consumed every day rich (including grains, vegetables, fruits, meats, eggs, dairy products, etc.)?	4.94	0.40	0.08	94.6	4.83	0.14	0.03	86.0	4.90	0.13	0.03	93.8
1.1.2 Do you regularly consume foods rich in protein (such as meat, fish, beans, etc.)?	4.86	0.12	0.02	86.4	4.82	0.12	0.02	85.8	4.80	0.10	0.02	85.5
1.1.3 Do you consume an adequate amount of vegetables and fruits?	4.90	0.05	0.01	93.7	4.93	0.08	0.02	95.3	4.89	0.15	0.03	89.0
1.1.4 Do you consume an appropriate amount of fats and sugars?	5.00	0.00	0.00	100.0	5.00	0.00	0.00	100.00	5.00	0.00	0.00	100.0
1.2.1 Is the intake amount of staple food for each meal appropriate?	4.83	0.11	0.02	86.0	4.86	0.12	0.02	86.5	4.82	0.14	0.00	85.9
1.2.2 Does the daily protein intake reach the recommended standard?	5.00	0.00	0.00	100.0	5.00	0.00	0.00	100.0	5.00	0.00	0.00	100.0
1.2.3 Does the daily intake of vegetables and fruits reach the recommended standard?	5.00	0.00	0.00	100.0	5.00	0.00	0.00	100.0	5.00	0.00	0.00	100.0
1.2.4 Is the daily water intake sufficient?	5.00	0.00	0.00	100.0	5.00	0.00	0.00	100.0	5.00	0.00	0.00	100.0
1.3.1 Do they have meals regularly every day (three meals at regular times)?	5.00	0.00	0.00	100.0	5.00	0.00	0.00	100.0	5.00	0.00	0.00	100.0
1.3.2 Do they have regular additional meals (if necessary)?	4.75	0.68	0.14	78.3	4.82	0.16	0.03	85.8	4.85	0.06	0.01	86.2
2.1.1 Do family members need to prepare meals for disabled older adults people?	3.56	0.81	0.23	53.00	3.54	0.43	0.12	52.0	3.47	0.68	0.20	0.40
2.1.2 Can family members provide comprehensive nutritional support?	4.94	0.15	0.03	95.4	4.95	0.13	0.03	95.5	4.85	0.12	0.02	86.3
2.1.3 Can family members adjust the diet according to the health condition of the older adults?	4.94	0.25	0.05	93.8	4.93	0.13	0.03	93.8	4.89	0.13	0.03	89.0
2.2.1 Have the caregivers received training in nutritional management?	4.94	0.25	0.05	93.8	4.83	0.26	0.05	86.0	4.88	0.10	0.02	87.0
2.2.2 Do the caregivers know how to identify and deal with the symptoms of malnutrition?	4.75	0.48	0.10	81.3	4.84	0.28	0.06	86.2	4.76	0.11	0.02	81.5
2.2.3 Do the caregivers know how to provide appropriate nutritional supplements for the older adults?	4.69	0.60	0.13	75.00	4.72	0.32	0.07	76.7	4.83	0.23	0.05	86.0
2.3.1 Can family members provide sufficient support and companionship?	4.91	0.10	0.02	93.8	4.90	0.10	0.02	93.7	4.86	0.18	0.04	86.4
2.3.2 Do family members pay attention to the eating habits and nutritional status of the older adults?	4.81	0.14	0.03	85.8	4.86	0.13	0.03	86.5	4.80	0.20	0.04	85.6
3.1.1 Is there a nutritional intervention plan for disabled older adults people?	4.88	0.25	0.05	87.0	4.92	0.08	0.02	94.1	4.90	0.06	0.01	93.6
3.1.2 Is there a specialized dietitian or doctor in charge of the nutritional management of the older adults?	4.88	0.15	0.03	87.0	4.83	0.16	0.03	86.0	4.86	0.13	0.03	86.4
3.2.1 Is personalized nutritional support provided according to the specific circumstances of the older adults?	4.81	0.20	0.04	81.3	4.80	0.33	0.07	85.5	4.83	0.15	0.03	86.0
3.2.2 Are there regular follow-ups and evaluations to ensure the effectiveness of the nutritional support?	4.83	0.15	0.03	86.0	4.79	0.23	0.05	80.2	4.83	0.14	0.03	86.0
3.2.3 Do you have any understanding of overnutrition?	3.53	0.65	0.18	51.8	3.48	0.53	0.15	49.8	3.50	0.53	0.15	51.0
3.3.1 Does the local medical institution have the ability and resources to provide comprehensive nutritional support?	4.90	0.35	0.07	93.6	4.86	0.21	0.04	86.5	4.83	0.16	0.03	86.0
3.3.2 Are there any nutritional education and publicity programs specifically for disabled older adults people?	4.85	0.23	0.05	86.2	4.83	0.13	0.03	86.0	4.86	0.21	0.04	86.4
4.1.1 Do psychological problems affect the eating habits and nutritional intake of the older adults?	4.95	0.03	0.01	95.6	4.90	0.05	0.01	93.8	4.89	0.14	0.03	93.5
4.1.2 Are there any intervention measures for psychological problems?	4.89	0.13	0.03	93.5	4.83	0.12	0.02	85.9	4.86	0.14	0.03	86.5
4.2.1 Can the older adults obtain sufficient social support (such as community services, volunteer assistance, etc.)?	4.83	0.22	0.05	86.0	4.93	0.14	0.03	93.8	4.89	0.14	0.03	87.1
4.2.2 Can social support help the older adults improve their diet management?	5.00	0.00	0.00	100.0	5.00	0.00	0.00	100.0	5.00	0.00	0.00	100.0
5.1.1 Have there been any fluctuations in the older adults’s BMI?	3.78	0.86	0.23	56.6	3.52	0.63	0.18	51.6	3.63	0.80	0.22	53.8
5.1.2 Are there any situations of malnutrition or overnutrition?	4.93	0.12	0.02	93.6	4.90	0.10	0.02	93.6	4.83	0.16	0.03	86.0
5.1.3 Are the blood test indicators (such as hemoglobin, albumin, etc.) normal?	4.86	0.23	0.05	86.4	4.89	0.24	0.05	93.5	4.85	0.14	0.03	86.2
5.2.1 Has the overall health condition of the older adults improved?	4.80	0.21	0.04	81.1	4.86	0.25	0.05	86.5	4.83	0.17	0.04	85.9
5.2.2 Has the number of complications related to malnutrition been reduced?	5.00	0.00	0.00	100.0	5.00	0.00	0.00	100.0	5.00	0.00	0.00	100.0
5.2.3 Has the quality of life of the older adults been improved?	5.00	0.00	0.00	100.0	5.00	0.00	0.00	100.0	5.00	0.00	0.00	100.0
5.3.1 Has the older adults’s ability for daily activities improved?	5.00	0.00	0.00	100.0	5.00	0.00	0.00	100.0	5.00	0.00	0.00	100.0
5.3.2 Can they perform daily activities (such as dressing, taking a bath, etc.) better?	5.00	0.00	0.00	100.0	5.00	0.00	0.00	100.0	5.00	0.00	0.00	100.0

According to the discussion, experts recommended necessary revisions to the indicator 2.1.1 “Do family members need to prepare meals for disabled older adults people?” to better align with clinical practice: 2.1.1 Do family members understand the basic nutritional needs of disabled older adults people? In addition, change “3.2.3 Do you have any understanding of overnutrition?” to “Is there an emergency plan for malnutrition?.” Change “5.1.1 Have there been any fluctuations in the older adults’s BMI?” to “5.1.1 Is the weight of the older adults stable?.” The reason for the change is that although BMI is more objective in evaluating weight, it involves calculations, which is not convenient for patients and caregivers to use. Therefore, weight is used instead of BMI.

In addition, “Does the medical institution provide regular nutritional assessment and guidance?” (Item 3.1.1) was added. The newly added indicator enriches the content of the family support system and medical support assessment, enabling the indicator system to more comprehensively cover the important aspects of the dietary management of disabled older adults people, and improving its integrity and scientificity. These significant modifications require a second-round validation.

### 3.3 Second round Delphi results

The second round of the Delphi method validated the tertiary indicators of the nutritional management indicator system for disabled older adults people. The appropriateness scores ranged from 4.85 to 4.94 (CV = 0.01–0.03; FR = 86.3–93.8%). The importance scores showed higher consistency (M = 4.85–4.94; CV = 0.01–0.04; FR = 86.2–93.9%), while the operational feasibility scores remained stable (M = 4.86–4.90; CV = 0.01–0.05; FR = 86.4–93.6%). The results indicate that these indicators do not require further revision (Table 6).

**Table 6:** Second-round expert consultation results for secondary indicators.

Secondary indicators	Applicability				Importance				Operational feasibility			
	M	SD	CV	FR (%)	M	SD	CV	FR (%)	M	SD	CV	FR (%)
2.1.1 Do family members understand the basic nutritional needs of disabled older adults people?	4.94	0.13	0.03	93.7	4.89	0.18	0.04	93.5	4.86	0.13	0.03	86.4
3.1.1 Does the medical institution provide regular nutritional assessment and guidance?	4.86	0.16	0.03	86.4	4.85	0.15	0.03	86.2	4.90	0.23	0.05	93.6
3.2.3 Is there an emergency plan for malnutrition?	4.85	0.12	0.02	86.3	4.86	0.13	0.03	86.5	4.89	0.10	0.02	93.5
5.1.1 Is the weight of the older adults stable?	4.91	0.06	0.01	93.8	4.94	0.03	0.01	93.9	4.90	0.03	0.01	93.6

Based on the two rounds of the Delphi method, the evaluation indicators for the dietary management of disabled older adults patients were finally determined (Supplementary Table S1).

## 4 Discussion

China has the largest older adults population globally, with 264 million people aged ≥60 years in 2020, accounting for 18.7% of the total population (2). This number is projected to reach 487 million by 2050, making aging a critical societal challenge (3). Among older adults, disability prevalence is rising: Wei et al. (40) have shown that 240 out of every 1,000 older people were disabled in 2019, translating to 63.4 million disabled older adult individuals. By 2050, this number is expected to exceed 95 million, driven by chronic diseases, age-related functional decline, and improved life expectancy (1). Disabled older adults face complex nutritional challenges, including medication-induced anorexia, dysphagia, and caregiver knowledge gaps (2). This study has constructed a comprehensive system through expert consultation, which includes 5 first-level items, 14 s-level items, and 39 third-level items. The system covers five key aspects: nutritional intake assessment, family care assessment, medical support assessment, psychological and social factor assessment, and nutritional management effectiveness assessment. This provides a valuable reference for the dietary management of disabled older adults people in China.

### 4.1 Nutritional intake assessment

The nutritional intake assessment highlights the critical gaps in protein and water intake among disabled older adult individuals. This finding aligns with global recommendations for sarcopenia prevention, as protein is essential for muscle synthesis, and inadequate intake increases sarcopenia risk (41). Water is equally important for metabolic functions, nutrient transport, and joint lubrication, and its insufficiency can indirectly affect muscle function (42). Given the physical limitations faced by disabled older adults people, such as chewing and swallowing difficulties, and illness-induced appetite loss, family caregivers play a pivotal role. They need professional nutrition training to effectively combine easily digestible protein-rich foods, master protein-boosting cooking skills, encourage regular water intake, and create personalized hydration plans. Medical institutions should provide nutritional guidance and prescribe supplements when necessary, while communities should organize nutritional science activities to raise public awareness, promote resource allocation, ensure adequate nutritional intake, reduce sarcopenia incidence, and enhance the quality of life for disabled older adult individuals.

### 4.2 Family care assessment

The family care assessment underscores the necessity of caregiver training, addressing the significant gap that 37.2% of Chinese family caregivers have not received formal education (43). Tang’s research showed that trained caregivers are better equipped to understand the special nutritional needs of disabled older adult individuals, make precise dietary adjustments based on specific health conditions, and master daily care skills to assist with safe physical activities. This not only relieves discomfort for bedridden patients but also reduces the risk of complications.

### 4.3 Medical support assessment

Medical support assessment aims to address the disparities in nutritional services between urban and rural areas. Urban medical institutions, equipped with advanced facilities can formulate personalized nutritional plans, offer face-to-face consultations, and provide comprehensive support for disabled older adult individuals with chronic diseases. In contrast, rural medical institutions often lack basic testing equipment, have few dietitians, and rely on general advice from grassroots doctors, which fails to meet complex nutritional needs (44, 45). To bridge this gap, institutional assessments should focus on identifying facility and talent shortages. The government must invest in rural areas to equip them with essential equipment. Establishing a support mechanism for urban–rural nutrition professionals, including training and knowledge exchange, is crucial.

### 4.4 Psychological and social factors assessment

Psychological and social factors assessment reveals the significant impact of depression on the dietary compliance of disabled older adult individuals. O’Keeffe et al. (46) confirmed a close correlation between malnutrition and psychological distress. Depression can lead to changes in appetite, severely disrupting dietary behavior and increasing the risk of malnutrition. Moreover, reduced social activities due to depression create a monotonous dietary environment, further diminishing the enthusiasm and regularity of eating (51). The lack of awareness of depression among families and society makes it difficult for patients to receive effective psychological intervention. To address these challenges, mental health professionals should provide active psychological counseling. Communities need to organize social activities to create a positive dietary atmosphere.

### 4.5 Nutritional management effectiveness assessment

Effect evaluation focuses on weight stability and functional improvement, aligning with national rehabilitation goals. Weight stability is crucial for the health of disabled older adult individuals, as it indicates balanced nutrition and metabolism (47). Through proper family care and medical support, an optimized diet with sufficient nutrients can minimize health risks associated with malnutrition or overnutrition (48). This boosts daily self-care abilities. To accurately evaluate these aspects, a scientific and comprehensive system is essential. Weight assessment should combine regular weighing with body composition analysis for a more precise nutritional profile.

In conclusion, the dietary management program developed in this study is scientifically valid and practically applicable. It provides a comprehensive framework for improving the nutritional status and quality of life of disabled older adult individuals in China. By addressing the multidimensional challenges faced by this vulnerable population, the program serves as a valuable tool for healthcare providers, policymakers, and caregivers.

### 4.6 Utility of structuring a dietary management programme for older adults people with disabilities

Proper dietary management is essential for addressing the multifaceted health challenges faced by older adult individuals with disabilities. The structured dietary management programme developed in this study has demonstrated significant utility in several key areas, as evidenced by the results obtained through the Delphi method.

### 4.7 Enhancing family care

The family care assessment revealed that caregivers play a pivotal role in the daily care of disabled older adult individuals. The programme emphasizes the need for caregiver training, which is crucial given that 37.2% of Chinese family caregivers have not received formal education (49). Trained caregivers are better equipped to understand the special nutritional needs of disabled older adult individuals and make precise dietary adjustments based on specific health conditions. This not only improves the quality of care but also enhances the overall wellbeing of the older adults. Future empirical validation of the relationships between indicators and outcomes is necessary. Clinical data (e.g., malnutrition incidence) and mixed methods should be introduced to reduce subjective bias.

### 4.8 Addressing psychological and social factors

The assessment of psychological and social factors underscores the impact of depression on dietary compliance. German et al. (50) has shown that depression can lead to changes in appetite and social withdrawal, both of which negatively affect nutritional intake. The programme’s inclusion of psychological support and social activities can help mitigate these effects. By integrating mental health interventions with nutritional support, the programme can improve both the physical and mental wellbeing of disabled older adult individuals.

### 4.9 Limitation

Although this study presents a validated set of indicators for evaluating the dietary management of disabled older adults patients, several limitations warrant consideration. First, the Delphi expert panel was composed solely of experts from tertiary hospitals in China, which may limit the generalizability of the research findings in resource - constrained settings such as community hospitals. Second, the Delphi method relies on expert judgment. While it has its advantages, it may be influenced by personal biases and knowledge gaps. Incorporating quantitative data and empirical research could provide additional validation for the indicator system. Third, the constructivist paradigm inherently favors expert consensus over predictive validity testing, so future empirical validation of the relationships between the indicators and outcomes is necessary. In the future, primary healthcare institutions, community caregivers, and interdisciplinary experts (such as rehabilitation therapists and psychologists) should be included to enhance the representativeness across different medical scenarios. Meanwhile, clinical data (such as the incidence of malnutrition and hospital outcomes) and mixed research methods (such as patient surveys) should be introduced to reduce reliance on subjective judgment.

## 5 Conclusion

This study represents one of the first efforts in China to systematically construct a dietary management indicator system for disabled older adults using a Delphi method. The resulting framework, developed through expert consensus, provides a theoretically grounded reference for clinical practice and future research. However, further empirical validation is needed to assess the system’s applicability and effectiveness in real-world settings. A total of three dimensions (health professionals involved in the treatment of the clinical practice guidelines for nutritional management of the older adults with disabilities, older adults with disabilities, and carers) were constructed. The management programme provides a reference for dietary management of the disabled older adults in China.

## Data Availability

The data analyzed in this study is subject to the following licenses/restrictions: the data used in this study will not be disclosed for the time being due to privacy concerns but are available from the corresponding author on reasonable request. Requests to access these datasets should be directed to ML, jmsdxlmy199219@163.com.

## References

[1] Knott RM Robertson M Muckersie E Forrester JOHNV. Glucose-mediated regulation of GLUT-1 and GLUT-3 mRNA in human retinal endothelial cells. Biochem Soc Trans. (1996) 24:216S. doi: 10.1042/bst024216s, 8736874

[2] Bayati M Arkia E Emadi M. Socio-economic inequality in the nutritional deficiencies among the world countries: evidence from global burden of disease study 2019. J Health Popul Nutr. (2025) 44:8. doi: 10.1186/s41043-025-00739-z, 39806471 PMC11731139

[3] Tang L Chen Y Wang F Liu Y Song Z Wang M . Safety and efficacy of day anterior cervical discectomy and fusion procedure for degenerative cervical spondylosis: a retrospective analysis. BMC Musculoskelet Disord. (2024) 25:223. doi: 10.1186/s12891-024-07356-7, 38504222 PMC10953196

[4] Su Z Hu Z Peng X. The impact of changes in China's family patterns on family pension functions. Int J Health Plann Manag. (2017) 32:351–62. doi: 10.1002/hpm.2436, 28736874

[5] Chen Z Park A. Rural pensions, intra-household bargaining, and elderly medical expenditure in China. Health Econ. (2023) 32:2353–71. doi: 10.1002/hec.4725 37418243

[6] Agarwal P Wang Y Buchman AS Bennett DA Morris MC. Dietary patterns and self-reported incident disability in older adults. J Gerontol A Biol Sci Med Sci. (2019) 74:1331–7. doi: 10.1093/gerona/gly211, 30247552 PMC6625581

[7] Cao XP Xu W Wang ZT Tan L Yu JT. Dietary components and nutritional strategies for dementia prevention in the elderly. Curr Alzheimer Res. (2023) 20:224–43. doi: 10.2174/1567205020666230609155932, 37303177

[8] McClinchy J. Dietary management of older people with diabetes. Br J Community Nurs. (2018) 23:248–51. doi: 10.12968/bjcn.2018.23.5.248 29708791

[9] Ghosh TS Rampelli S Jeffery IB Santoro A Neto M Capri M . Mediterranean diet intervention alters the gut microbiome in older people reducing frailty and improving health status: the NU-AGE 1-year dietary intervention across five European countries. Gut. (2020) 69:1218–28. doi: 10.1136/gutjnl-2019-319654, 32066625 PMC7306987

[10] DamiÃ R Meneguci J da Silva Santos Á Matijasevich A Menezes PR. Nutritional risk and quality of life in community-dwelling elderly: a cross-sectional study. J Nutr Health Aging. (2018) 22:111–6. doi: 10.1007/s12603-017-0935-y, 29300430 PMC12880387

[11] Adıgüzel E Acar-Tek N. Nutrition-related parameters predict the health-related quality of life in home care patients. Exp Gerontol. (2019) 120:15–20. doi: 10.1016/j.exger.2019.02.018, 30822485

[12] Maseda A Diego-Diez C Lorenzo-López L López-López R Regueiro-Folgueira L Millán-Calenti JC. Quality of life, functional impairment and social factors as determinants of nutritional status in older adults: the VERISAÚDE study. Clin Nutr. (2018) 37:993–9. doi: 10.1016/j.clnu.2017.04.009 28456537

[13] Ferguson CC Clina JG Young HJ Gammon L Jeter A Abadie A . Improvements in nutrition knowledge among adults with physical disabilities: a program evaluation of the mindfulness, exercise, and nutrition to optimize resilience (MENTOR) program 2020-2021. Disabil Health J. (2024) 17:101577. doi: 10.1016/j.dhjo.2023.101577 38184471

[14] Penfold CN Evans BT. Giant cell lesions complicating Paget's disease of bone and their response to calcitonin therapy. Br J Oral Maxillofac Surg. (1993) 31:267. doi: 10.1016/0266-4356(93)90158-s 8399049

[15] Duran-Poveda M Jimenez-Fonseca P Sirvent-Ochando M García-Luna PP Pereira-Cunill JL Lema-Marqués B . Integral nutritional approach to the care of cancer patients: results from a Delphi panel. Clin Transl Oncol. (2018) 20:1202–11. doi: 10.1007/s12094-018-1846-z 29500682

[16] Hironaka S Kugimiya Y Watanabe Y Motokawa K Hirano H Kawai H . Association between oral, social, and physical frailty in community-dwelling older adults. Arch Gerontol Geriatr. (2020) 89:104105. doi: 10.1016/j.archger.2020.104105 32480111

[17] Kaur D Rasane P Singh J Kaur S Kumar V Mahato DK . Nutritional interventions for elderly and considerations for the development of geriatric foods. Curr Aging Sci. (2019) 12:15–27. doi: 10.2174/1874609812666190521110548 31109282 PMC6971894

[18] Li Z Zhang L Yang Q Zhou X Yang M Zhang Y . Association between geriatric nutritional risk index and depression prevalence in the elderly population in NHANES. BMC Public Health. (2024) 24:469. doi: 10.1186/s12889-024-17925-z, 38355455 PMC10868080

[19] Otsuka R. Nutrition for older adults. J Nutr Sci Vitaminol. (2022) 68:S61–3. doi: 10.3177/jnsv.68.S61 36437020

[20] Hallit S Daher MC Hallit R Hachem D Kheir N Salameh P. Correlates associated with mental health and nutritional status in Lebanese older adults: a cross-sectional study. Arch Gerontol Geriatr. (2020) 87:103879. doi: 10.1016/j.archger.2019.05.004, 31160065

[21] McClung HL Ptomey LT Shook RP Aggarwal A Gorczyca AM Sazonov ES . Dietary intake and physical activity assessment: current tools, techniques, and Technologies for use in adult populations. Am J Prev Med. (2018) 55:e93–e104. doi: 10.1016/j.amepre.2018.06.011 30241622

[22] Wickramasinghe K Mathers JC Wopereis S Marsman DS Griffiths JC. From lifespan to healthspan: the role of nutrition in healthy ageing. J Nutr Sci. (2020) 9:e33. doi: 10.1017/jns.2020.26, 33101660 PMC7550962

[23] An R Chiu CY Andrade F. Nutrient intake and use of dietary supplements among US adults with disabilities. Disabil Health J. (2015) 8:240–9. doi: 10.1016/j.dhjo.2014.09.001, 25306424

[24] An R Chiu CY Zhang Z Burd NA. Nutrient intake among US adults with disabilities. J Hum Nutr Diet. (2015) 28:465–75. doi: 10.1111/jhn.12274 25233949

[25] Wei C Wagler JB Rodrigues IB Giangregorio L Keller H Thabane L . Telephone Administration of the Automated Self-Administered 24-hour dietary assessment in older adults: lessons learned. Can J Diet Pract Res. (2022) 83:30–4. doi: 10.3148/cjdpr-2021-024, 34582280

[26] Jang K. An observational study on the association between nutritional intake and mental health among older adults in rural areas. Nurs Health Sci. (2025) 27:e70080. doi: 10.1111/nhs.70080 40101703 PMC11919469

[27] Kwak Y Kim Y. Association between mental health and meal patterns among elderly Koreans. Geriatr Gerontol Int. (2018) 18:161–8. doi: 10.1111/ggi.13106, 28675623

[28] Ghimire S Baral BK Karmacharya I Callahan K Mishra SR. Life satisfaction among elderly patients in Nepal: associations with nutritional and mental wellbeing. Health Qual Life Outcomes. (2018) 16:118. doi: 10.1186/s12955-018-0947-2, 29880002 PMC5992629

[29] Krok-Schoen JL Jonnalagadda SS Luo M Kelly OJ Taylor CA. Nutrient intakes from meals and snacks differ with age in middle-aged and older Americans. Nutrients. (2019) 11:301. doi: 10.3390/nu11061301, 31181765 PMC6627320

[30] Sun Q Jiang N Lu N Lou VWQ. Bidirectional relationship between cognitive function and loss hierarchy of activities of daily living among older adults with disabilities in urban China: a cross-lagged analysis. BMJ Open. (2022) 12:e057211. doi: 10.1136/bmjopen-2021-057211, 36691162 PMC9442490

[31] Lengyel CO Jiang D Tate RB. Trajectories of nutritional risk: the Manitoba follow-up study. J Nutr Health Aging. (2017) 21:604–9. doi: 10.1007/s12603-016-0826-7 28537322 PMC12879859

[32] Pilleron S Pérès K Jutand MA Helmer C Dartigues JF Samieri C . Dietary patterns and risk of self-reported activity limitation in older adults from the Three-City Bordeaux study. Br J Nutr. (2018) 120:549–56. doi: 10.1017/S0007114518001654, 29987992

[33] Mendonca N Hill TR Granic A Davies K Collerton J Mathers JC . Macronutrient intake and food sources in the very old: analysis of the Newcastle 85+ study. Br J Nutr. (2016) 115:2170–80. doi: 10.1017/S0007114516001379, 27087119

[34] Lee RE O’Neal A Cameron C Hughes RB O’Connor DP Ohri-Vachaspati P . Developing content for the food environment assessment survey tool (FEAST): a systematic mixed methods study with people with disabilities. Int J Environ Res Public Health. (2020) 17:781. doi: 10.3390/ijerph17217781, 33114296 PMC7660641

[35] Meskell P Murphy K Shaw DG Casey D. Insights into the use and complexities of the policy Delphi technique. Nurse Res. (2014) 21:32–9. doi: 10.7748/nr2014.01.21.3.32.e342, 24460564

[36] Latif RA Mohamed R Dahlan A Mat Nor MZ. Using Delphi technique: making sense of consensus in concept mapping structure and multiple choice questions (MCQ). Educ. Med. J. (2016) 8:421. doi: 10.5959/eimj.v8i3.421

[37] Gnatzy T Warth J von der Gracht H Darkow IL. Validating an innovative real-time Delphi approach - a methodological comparison between real-time and conventional Delphi studies. Technol. Forecasting Soc. Change. (2011) 78:1681–94. doi: 10.1016/j.techfore.2011.04.006

[38] Braun V Clarke V. Reflecting on reflexive thematic analysis. Q. Res. Sport. (2019) 11:589–97. doi: 10.1080/2159676X.2019.1628806

[39] Byrne D. A worked example of Braun and Clarke's approach to reflexive thematic analysis. Qual Quant. (2021) 56:1391–412. doi: 10.1007/s11135-021-01182-y

[40] Wei L Yi H Hai-Lu Z. Elderly people with disabilities in China. J Am Geriatr Soc. (2019) 67:858–9. doi: 10.1111/jgs.15793 30720879

[41] Robinson SM Reginster JY Rizzoli R Shaw SC Kanis JA Bautmans I . Does nutrition play a role in the prevention and management of sarcopenia? Clin Nutr. (2018) 37:1121–32. doi: 10.1016/j.clnu.2017.08.016, 28927897 PMC5796643

[42] Popkin BM D'Anci KE Rosenberg IH. Water, hydration, and health. Nutr Rev. (2010) 68:439–58. doi: 10.1111/j.1753-4887.2010.00304.x, 20646222 PMC2908954

[43] Guanxiu T Chunli Y Bing F Meili X Pingping Y Jun L. Training effect of family caregivers for disabled elderly people based on the online care knowledge and skill system. Chin J Modern Nurs. (2019) 25:7–10. doi: 10.3760/CMA.J.ISSN.1674-2907.2019.01.002

[44] Swift EK. Guidance for the National Healthcare Disparities Report. Washington (DC): National Academies Press (2002). 25057628

[45] Shavers VL. Measurement of socioeconomic status in health disparities research. J Natl Med Assoc. (2007) 106:792–8. doi: 10.1016/S0929-6646(08)60043-1 PMC2575866 17913111

[46] O'Keeffe M Kelly M O'Herlihy E O'Toole PW Kearney PM Timmons S . Potentially modifiable determinants of malnutrition in older adults: a systematic review. Clin Nutr. (2019) 38:2477–98. doi: 10.1016/j.clnu.2018.12.007, 30685297

[47] Monteverde M. Excess weight and disability among the elderly in Argentina. Salud Colect. (2015) 11:509–21. doi: 10.18294/sc.2015.792, 26676594

[48] Fitryasari R Yusuf A Nursalam Tristiana RD Nihayati HE. Family members' perspective of family resilience's risk factors in taking care of schizophrenia patients. Int J Nurs Sci. (2018) 5:255–61. doi: 10.1016/j.ijnss.2018.06.002, 31406834 PMC6626218

[49] Yue L Jia C Hu B Zhang Z Bai M Wang S . Caregiving stress among family caregivers of older adults living with disabilities in China. Geriatr Nurs. (2022) 47:226–31. doi: 10.1016/j.gerinurse.2022.07.017 35987148

[50] German L Kahana C Rosenfeld V Zabrowsky I Wiezer Z Fraser D . Depressive symptoms are associated with food insufficiency and nutritional deficiencies in poor community-dwelling elderly people. J Nutr Health Aging. (2011) 15:3–8. doi: 10.1007/s12603-011-0005-9, 21267514 PMC12879720

[51] Konttinen H van Strien T Männistö S Jousilahti P Haukkala A. Depression, emotional eating and long-term weight changes: a population-based prospective study. Int J Behav Nutr Phys Act. (2019) 16:28. doi: 10.1186/s12966-019-0791-8, 30894189 PMC6427874

