# Supplementary material for: Development of a dietary management indicator system for older adults with disabilities: a Delphi study
Source: Front Public Health. 2026 Mar 23;14:1796150. doi: 10.3389/fpubh.2026.1796150 (PMC13050889; doi:10.3389/fpubh.2026.1796150)
Supplement: Supplementary file 1 [file Table_1.DOCX]

Table S1 Construction of Sensitive Indicators

| Primary indicators | Secondary indicators | Tertiary indicators |
| --- | --- | --- |
| 1 Assessment of Nutritional Intake | 1.1 Types of food | 1.1.1 Is the variety of foods consumed every day rich (including grains, vegetables, fruits, meats, eggs, dairy products, etc.)? |
|  |  | 1.1.2 Do you regularly consume foods rich in protein (such as meat, fish, beans, etc.)? |
|  |  | 1.1.3 Do you consume an adequate amount of vegetables and fruits? |
|  |  | 1.1.4 Do you consume an appropriate amount of fats and sugars? |
|  | 1.2Intake amount | 1.2.1Is the intake amount of staple food for each meal appropriate? |
|  |  | 1.2.2 Does the daily protein intake reach the recommended standard? |
|  |  | 1.2.3 Does the daily intake of vegetables and fruits reach the recommended standard? |
|  |  | 1.2.4Is the daily water intake sufficient? |
|  | 1.3Eating frequency | 1.3.1 Do they have meals regularly every day (three meals at regular times)? |
|  |  | 1.3.2 Do they have regular additional meals (if necessary)? |
| 2 Assessment of Family Care | 2.1Family diet arrangement | 2.1.1 Do family members understand the basic nutritional needs of disabled elderly people? |
|  |  | 2.1.2 Can family members provide comprehensive nutritional support? |
|  |  | 2.1.3 Can family members adjust the diet according to the health condition of the elderly? |
|  | 2.2 The knowledge and skills of caregivers | 2.2.1 Have the caregivers received training in nutritional management? |
|  |  | 2.2.2 Do the caregivers know how to identify and deal with the symptoms of malnutrition? |
|  |  | 2.2.3 Do the caregivers know how to provide appropriate nutritional supplements for the elderly? |
|  | 2.3 Family support system | 2.3.1 Can family members provide sufficient support and companionship? |
|  |  | 2.3.2 Do family members pay attention to the eating habits and nutritional status of the elderly? |
|  |  | 2.3.3 Patients who are unable to eat properly are dependent on nasal or parenteral nutrition. |
| 3 Assessment of Medical Support | 3.1 Medical Intervention | 3.1.1 Does the medical institution provide regular nutritional assessment and guidance? |
|  |  | 3.1.2 Is there a nutritional intervention plan for disabled elderly people? |
|  |  | 3.1.3 Is there a specialized dietitian or doctor in charge of the nutritional management of the elderly? |
|  | 3.2 Implementation of Nutritional Support | 3.2.1 Is personalized nutritional support provided according to the specific circumstances of the elderly? |
|  |  | 3.2.2 Are there regular follow-ups and evaluations to ensure the effectiveness of the nutritional support? |
|  |  | 3.2.3 Is there an emergency plan for malnutrition? |
|  | 3.3 The accessibility of medical resources | 3.3.1 Does the local medical institution have the ability and resources to provide comprehensive nutritional support? |
|  |  | 3.3.2 Are there any nutritional education and publicity programs specifically for disabled elderly people? |
| 4. Assessment of Psychological and Social Factors | 4.1 Mental condition | 4.1.1 Are there any psychological problems such as depression and anxiety among the elderly? |
|  |  | 4.1.2 Do psychological problems affect the eating habits and nutritional intake of the elderly? |
|  |  | 4.1.3 Are there any intervention measures for psychological problems? |
|  | 4.2 Social Support | 4.2.1Can the elderly obtain sufficient social support (such as community services, volunteer assistance, etc.)? |
|  |  | 4.2.2Can social support help the elderly improve their diet management? |
| 1. Evaluation of the Effectiveness of Nutritional Management | 5.1 Nutritional status | 5.1.1 Is the weight of the elderly stable? |
|  |  | 5.1.2 Are there any situations of malnutrition or overnutrition? |
|  |  | 5.1.3 Are the blood test indicators (such as hemoglobin, albumin, etc.) normal? |
|  | 5.2 Health condition | 5.2.1 Has the overall health condition of the elderly improved? |
|  |  | 5.2.2Has the number of complications related to malnutrition been reduced? |
|  |  | 5.2.3 Has the quality of life of the elderly been improved? |
|  | 5.3 Functional status | 5.3.1 Has the elderly's ability for daily activities improved? |
|  |  | 5.3.2 Can they perform daily activities (such as dressing, taking a bath, etc.) better? |
